# Gender Differences in Neuromuscular Control during the Preparation Phase of Single-Leg Landing Task in Badminton

**DOI:** 10.3390/jcm12093296

**Published:** 2023-05-05

**Authors:** Zhe Hu, Yanan Zhang, Tengfei Dong, Maolin Dong, Sukwon Kim, Youngsuk Kim

**Affiliations:** Department of Physical Education, Jeonbuk National University, Jeonju 54896, Republic of Korea

**Keywords:** knee, EMG, muscle, ACL, badminton player, injury prevention

## Abstract

Background: Studies on the biomechanical mechanisms of an anterior cruciate ligament (ACL) injury have been extensively studied, but studies on the neuromuscular control-related risk factors for an ACL injury in specific maneuvers have not been reported for badminton players. Study design: Controlled laboratory study. Methods: Sixteen badminton players (8 male, 8 female) performed a single-leg badminton ball landing task, and lower limb muscle activity, kinematic data, and ground reaction force were measured during this procedure using marker-based movement analysis, force plates, and electromyography (EMG). Gender differences in the lower limb kinematic data, mean values of normalized lower limb muscle activation (MVC%), and co-contraction values during the landing preparation phase (100 ms before initial contact) were analyzed using MANOVA. Results: In the badminton landing task, the knee valgus angle was greater in females than in males (6.27 ± 2.75 vs. 1.72 ± 3.20) in the pre-landing preparation position. Compared to male badminton players, females exhibited greater gluteus maximus (44.92 ± 18.00 vs. 20.34 ± 11.64), rectus femoris (41.56 ± 9.84 vs. 26.14 ± 10.46), and medial gastrocnemius (37.39 ± 17.31 vs. 19.11 ± 11.17) lateral gastrocnemius (36.86 ± 17.82 vs. 13.59 ± 2.71) muscle activity (MVC%). Conclusion: Female badminton players exhibit neuromuscular control strategies that may be inadequate for ACL protection and may be a potential risk factor for a high incidence of ACL injury In the future, when devising injury prevention plans for female badminton players, optimizing neuromuscular control during the pre-landing phase can be targeted.

## 1. Introduction

Badminton is one of the most popular sports in the world with approximately 200 million participants [1]. Despite its popularity, there is relatively little research on badminton injuries. Epidemiological reports on injuries among badminton players indicate that the lower extremities are the most commonly injured body part, accounting for about 70% of all injuries. More than two-thirds of serious injuries (requiring surgery) occur in the knee joint, including ligament tears (71.5%), cartilage tears (20.3%), and fractures (2.6%) [2]. Anterior cruciate ligament (ACL) injuries usually occur in non-contact activities that require repeated jumping, landing, and changes in direction. Due to the nature of badminton, ACL injuries also affect badminton players, especially female players with a 2.5 to 4.8 times higher risk than that of male players [3,4]. Once an ACL injury occurs, it not only requires costly surgery and extensive rehabilitation, but also increases the risk of early osteoarthritis of the knee joint [5]. Given the serious consequences of such injuries, it is crucial to identify the exact mechanisms behind them to effectively prevent them.

The gender differences in non-contact cruciate ligament injuries have been attributed to several factors [6,7,8], including anatomical factors, hormone levels, and neuromuscular control. The high incidence of anterior cruciate ligament injuries is thought to be related to the large loads generated in the knee joint. However, reasons for this knee-loading pattern in females are not fully understood. The investigation of neuromuscular control factors has become an interesting avenue of research, as joint kinematics and moments are controlled by the muscle tissue surrounding the joint. In previous studies, large anterior tibial shear forces, extension moments, valgus moments, valgus angles, and small extension angles of the knee have been considered risk factors for ACL injury. Poor neuromuscular control may cause the development of risk factors that lead to subsequent ACL injuries [9,10,11]. In studies addressing lower extremity muscle activity patterns, there were gender differences in neuromuscular control between males and females during the performance of the landing task. Females exhibit stronger quadriceps activity relative to its antagonist muscle (hamstring), and this quadriceps activation strategy may increase knee anterior shear forces at smaller knee angles [12,13,14]. Similarly, increased or unbalanced gastrocnemius activity in females compared to males during the lateral cutting task puts the knee in an unfavorable position, and this activation strategy may result in greater stress and strain in the ACL [10,12,15]. In addition, gender differences in the control of gluteal muscle (gluteus maximus and gluteus medius) activity in females, compared to males, may increase the likelihood of frontal plane instability in the knee joint, placing increased knee loading, and thus increasing the risk of ACL injury [16]. Furthermore, in studies targeting co-contraction, the co-activation strategies (anterior-posterior and medial-lateral lower extremity muscles) exhibited by females may not be sufficient to generate sufficient stiffness to maintain knee sagittal and frontal plane stability, which reduces the generation of load at the knee joint and would also adversely affect ACL injury. It is worth noting, however, that although some studies have explored neuromuscular control as a risk factor for non-contact ACL injury during landing tasks, there is specificity in the movements of ACL injury in different sport populations and the sport itself, for example, volleyball is a jump landing movement [17], soccer is a cutting landing movement [15], and badminton is a posterior lateral single-leg landing maneuver [18]. This study aims to focus on badminton and compare the electromyography (EMG) signals and 3D kinematics of female and male badminton players during the high-risk single-leg landing maneuver in an environment as close to the playing field as possible. By combining EMG and kinematics, the study explores the neuromuscular control factors that may contribute to the high incidence of ACL injury in females and provides information for the prevention of an ACL injury.

In summary, this study aimed to compare the EMG signals and three-dimensional kinematics of female and male badminton players during the single-leg landing maneuver.

## 2. Materials and Methods

### 2.1. Subjects

A total of 16 badminton players, including 8 males and 8 females, were recruited to participate in this study. Females were 21.50 (±2.45) years old, 1.67 (±0.05) m tall, and 59.50 (±5.71) kg in mass, whereas males were 20.63 (±0.92) years old, 1.78 (±0.03) m tall, and 71.63 (±9.97) kg in mass. All participants were recruited by Jeonbuk University and the inclusion criteria were: (1) an experienced physiotherapist, to determine via observation and brief assessment, that there is no significant restriction of movement or muscle weakness; (2) no lower extremity pain before the test; and (3) subjects are required to participate in organized training at least four times a week. For the standardized test, the subjects were selected as badminton players with the right hand as the dominant hand. This study was approved by the Ethics Committee of Jeonbuk University (JBNU2022-01-004-002). Before participation in this study, all subjects were informed of the trial procedures and read and signed the informed consent form.

### 2.2. Prepare for Testing

**Figure 1:**
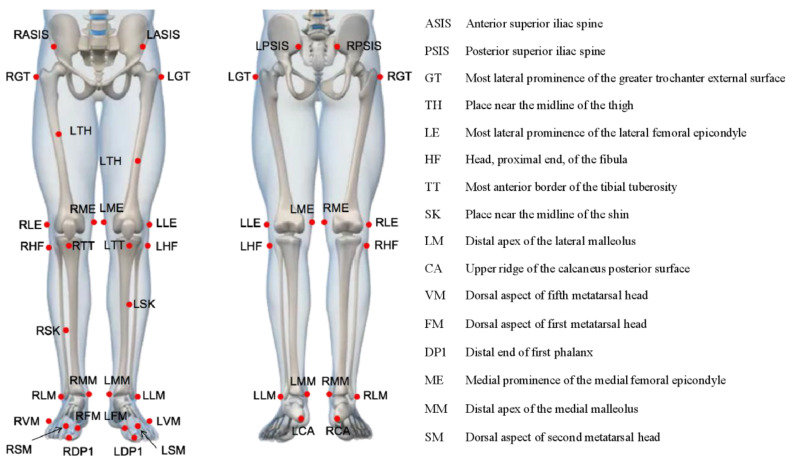
Anatomical position of the participant’s pelvis and lower extremities for placement of reflex markers (*N* = 32). The “R” and “L” represent the right and left sides, respectively.

We collected trial data using 13 infrared cameras (OptiTrack, LEYARD, Buffalo Grove, IL, USA) to capture the kinematic data of each participant. The cameras had a sampling rate of 120 Hz. The Rizzoli Lower Body protocol integrates a novel marker placement for lower body tracking. This marker set is designed to provide a complete description of the 3D segment and joint motion for analyzing the pelvis and lower extremity kinematics [19,20]. Retro reflective markers (*N* = 32) are located in the anatomical landmarks as shown in Figure 1. 

Ground reaction force data were collected at 1200 Hz using an OR6-6-2000 force platform (AMTI Inc.) from Newton, Maryland, USA with a maximum delay time of 6 ms.

For the EMG signal acquisition, we used a Trigno Avanti sensor (Delsys, Natick, MA, USA; 3.7 cm × 2.7 cm). All EMG sensors (Trigno Avanti Sensor) had a common-mode rejection ratio of 80 dB and were synchronized with kinematic and kinetic data by recording software (OptiTrack, LEYARD, USA) and EMG was sampled at 1200 Hz. Surface electrodes were selected from gluteus maximus (GMAX), gluteus medius (GMED), rectus femoris (RF), medial hamstrings (semitendinosus, MH), lateral hamstrings (biceps femoris, LH), medial gastrocnemius (MG), and lateral gastrocnemius (LG). The choice of the position of each muscle EMG, and how the maximum voluntary isometric contraction was tested, is shown in Table 1 [21].

The hair on the skin surface was shaved and cleaned with alcohol before the electrodes were attached. After the skin was dry, the EMG electrodes were attached. At the same time, motion tape was used to fix the electrodes and reduce motion artifacts [22]. The maximum voluntary isometric contraction (MVC) test was performed on each muscle for 5 s in the following manner (Table 1).

Fengcai’s badminton server SPT6000 (SPTLOOKER, Guangzhou, China) was used to send the shuttlecock to the designated area in the same state. Subjects wear uniform shorts, individual socks, and shoes, and use uniform rackets.

### 2.3. Test Procedure

**Figure 2:**
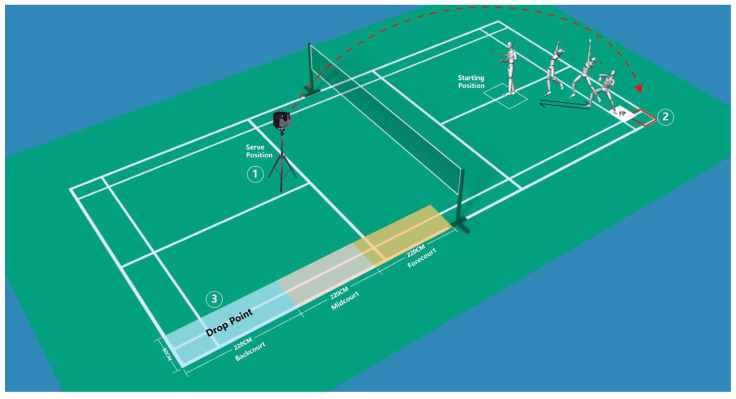
Experimental setup. Force plate (FP) and badminton serve machine position. The badminton serve machine sends shuttlecocks from area ① to area ② which is 50 cm × 50 cm. The red arrow represents the trajectory of badminton. The subject steps back from the starting point in a left–back direction, then jumps and performs an overhead strike. The subject performs a single-leg landing on the force plate and then returns to the starting position. Area ③ is the shuttlecock drop point after hitting the shuttlecock.

The laboratory design is shown in Figure 2.

The badminton serve position ① is located at the intersection of the center line and the serve line, and the badminton is sent to the designated area ② (50 cm × 50 cm) in the same state through the badminton serve machine, as referred to by our previous research [23].

**Figure 3:**
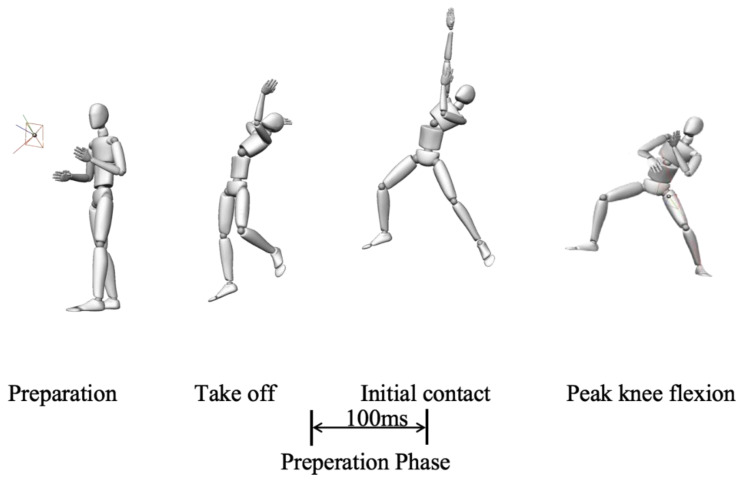
Events of badminton single-leg landing task.

Subjects performed a 10 min warm-up (jogging or swinging), and then performed the single-leg landing maneuver test after a backhand side overhead stroke, which is considered to be the maneuver with the highest incidence of ACL injuries [18]. A badminton coach with around 10 years of experience as a competitive player demonstrated the footwork and overhead stroke task to each of the subjects. Starting from the starting position, subjects simulate a backhand side step to the left rear of the court, and after performing an overhead stroke, the left leg lands on the force plate and they quickly return to the starting position. Subjects simply hit the shuttlecock in their customary manner to the opposite back side of the court area ③ (220 cm × 80 cm). Subjects were allowed to perform several exercises, followed by three to five consecutive trials. The main maneuvers are shown in Figure 3.

### 2.4. Data Processing and Analysis

The kinematic data were processed by Visual 3d (C-Motion, Inc., Germantown, MD, USA). The pelvis was defined relative to the global (laboratory) coordinate system and assigned six (three translational and three rotational) degrees of freedom. Using the coda model approach, the hip center is defined by the right and left anterior superior iliac spine and the right and left posterior superior iliac spine, the knee center by the medial femoral epicondyle and the lateral femoral epicondyle, and the ankle center by the medial and lateral ankle [23]. The definition of the directions for the segments of the pelvis, thigh, and leg is as follows: the positive *Y*-axis points forward; the positive *X*-axis points inward; and the positive *Z*-axis points upward. The angle of the hip joint is defined as the thigh’s angle relative to the pelvis, the angle of the knee joint is defined as the leg’s angle relative to the thigh, and the angle of the ankle joint is defined as the foot’s angle relative to the leg. The motion of the hip and knee joints is defined as flexion/extension in the sagittal plane of the medial-lateral *X*-axis, adduction/abduction in the coronal plane of the anterior-posterior *Y*-axis, and internal/external rotation in the horizontal plane of the vertical *Z*-axis. The motion of the ankle joint is defined as dorsiflexion and plantarflexion in the sagittal plane of the medial-lateral *X*-axis, inversion/eversion in the coronal plane of the anterior-posterior *Y*-axis, and abduction/adduction in the horizontal plane of the vertical *Z*-axis with the direction determined by the right-hand screw rule. By setting the positive and negative signs, the hip and knee directions are unified as positive for flexion, negative for extension, positive for abduction, negative for adduction, positive for internal rotation, and negative for external rotation. It should be noted that in ankle joint angles, the angle of 90 degrees between the foot and the sagittal plane of the lower leg is defined as 0 degrees in anatomical standing, dorsiflexion (positive) for upward and plantarflexion (negative) for downward, eversion for positive, inversion for negative, adduction was positive and abduction was negative.

We mainly processed and analyzed the muscle activity and co-contraction activity, as well as kinematic data during the pre-landing preparation phase. The pre-landing preparation phase was defined as the 100 milliseconds before initial contact (IC) with the force plate, as this phase is considered to reflect the pre-activation status of the muscles before landing [24,25]. The IC moment was defined as the first frame in which the force plate data exceeded 10 N. We collated the kinematic data from 1 hz before the initial contact moment as the kinematic data of the pre-landing preparation phase. To process and filter the raw data, both isometric and dynamic tests underwent filtering using a fourth-order Butterworth bandpass filter with a cut-off frequency range of 10–400 Hz. The signal was then smoothed using a root mean square (RMS) algorithm with a window size of 0.04 s and an overlap of 0.02 s between windows. To make the content easier to understand, we first determined the average RMS amplitude for each muscle during the preparation phase before landing on a single leg. Then, we normalized these values by comparing them to the RMS values obtained during the MVC for each muscle. The hamstring co-contraction ratio (M/LHAM) was calculated as the mean of MHAM RMS divided by the mean of LHAM RMS [15]. The gastrocnemius co-contraction ratio (M/LGAS) was calculated as the mean of MGAS RMS divided by the mean of LGAS RMS [15]. The hamstring/quadriceps co-contraction ratio (H/Q) was calculated as the mean of the sum of MHAM RMS and LHAM RMS activity divided by the mean of RF RMS activity [26]. The gastrocnemius/quadriceps co-contraction ratio (GAS/Q) was calculated as the mean of the sum of MGAS RMS and LGAS RMS activity divided by the mean of RF RMS activity [9].

Statistical analysis. Multivariate analysis of variance (MANOVA) was used to test the statistical differences between the male and female badminton player groups. The outcome variables were as follows: normalized EMG activity of lower limb muscles (MVC%); co-contraction index of lower limb muscles; and hip-knee-ankle joint angle. Statistical analyses were performed using SPSS 26.0 software (SPSS for Windows, Chicago, IL, USA) with a significance level of *p* < 0.05.

## 3. Results

**Figure 4:**
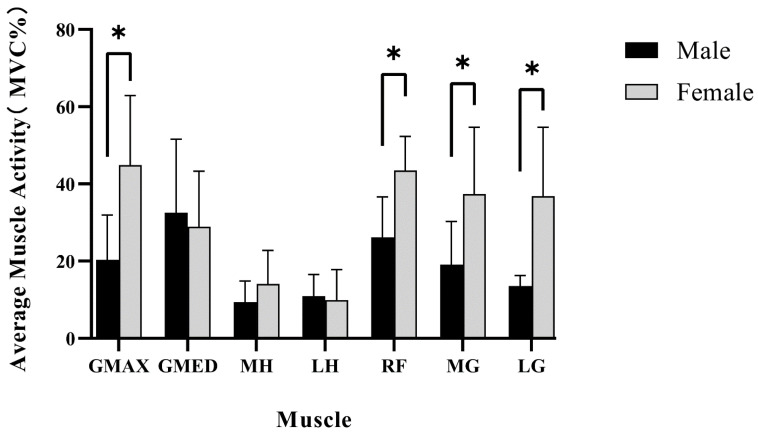
Mean and standard deviation of lower limb muscle activity during the preparation phase before landing for the single-leg landing task in badminton. * Represents statistically significant differences. GMAX: gluteus maximus, GMED: gluteus medius, RF: rectus femoris, MH: medial hamstrings (semitendinosus), LH: lateral hamstrings (biceps femoris), MG: medial gastrocnemius, and LG: lateral gastrocnemius.

The mean and standard deviation of lower limb muscle activity during the landing preparation phase of a single-leg landing task after a backhand side overhead stroke in badminton, as shown in Figure 4.

In the badminton single-leg landing task, during the landing preparation phase, the normalized muscle activity values of gluteus maximus were 44.92 ± 18.00 in female badminton players and 20.34 ± 11.64 in males. The activity of the gluteus maximus muscle in female badminton players was 24.58% higher than that of males and there was a significant gender difference (*p* < 0.05). The normalized rectus femoris muscle activity values were 41.56 ± 9.84 in female badminton players and 26.14 ± 10.46 in males. The activity of the rectus femoris muscle was 15.42% higher in female badminton players than in males, and thus, there was a significant gender difference (*p* < 0.05). The normalized medial gastrocnemius muscle activity values were 37.39 ± 17.31 in female badminton players and 19.11 ± 11.17 in males. The activity of the medial gastrocnemius muscle was 18.28% higher in female badminton players than in males, and thus, there was a significant gender difference (*p* < 0.05). The normalized muscle activity value of the lateral gastrocnemius was 36.86 ± 17.82 in female badminton players, compared to 13.59 ± 2.71 in males. The activity of the lateral gastrocnemius muscle was 23.27% higher in female badminton players than in males, and thus, there was a significant gender difference (*p* < 0.05).

**Figure 5:**
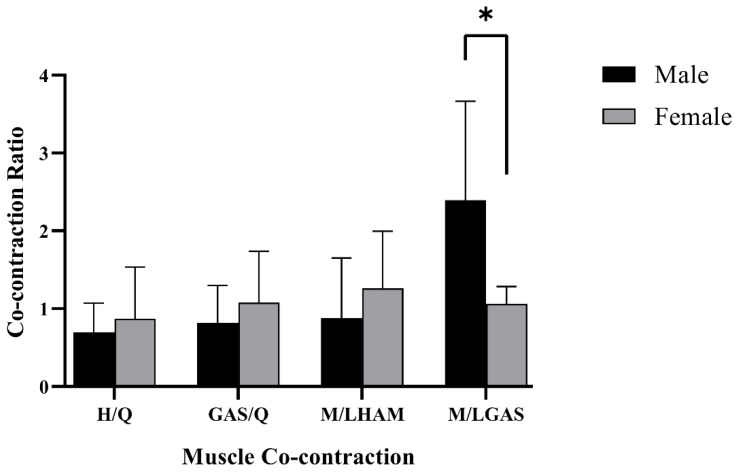
Badminton single-leg landing task, mean and standard deviation of lower limb muscle co-contraction activity ratio during the preparation phase before landing. * Represents statistically significant differences. H/Q: hamstring/quadriceps co-contraction ratio, M/LHAM: medial/lateral hamstring co-contraction ratio, M/LGAS: medial/lateral gastrocnemius co-contraction ratio, GAS/Q: gastrocnemius/quadriceps co-contraction ratio.

In the single-leg landing task of badminton, the gender differences in lower limb muscle co-contractions during the pre-landing preparation phase are shown in Figure 5.

The ratio of medial and lateral gastrocnemius co-contractions was 1.06 ± 0.22 in female badminton players and 2.39 ± 1.27 in males, which is 1.33 higher in males than females, and thus, there was a significant gender difference (*p* < 0.05).

The gender differences in the lower limb kinematic data of male and female badminton players in the landing preparation position are shown in Table 2. 

The knee valgus angle for female badminton players was (6.27 ± 2.75) degrees compared to (1.72 ± 3.20) degrees for males. The valgus angle was 5.5 degrees higher than among male players, and thus, there was a significant difference (*p* < 0.05).

## 4. Discussion

The results of this study showed that female badminton players exhibited kinematic characteristics combining muscle activity strategies of the large gluteus maximus, rectus femoris, and gastrocnemius muscles and co-activation strategies of the large external medial gastrocnemius muscles during the landing preparation phase compared to males. These gender biases in neuromuscular control appear to place females at greater risk of cruciate ligament injury.

The gluteus maximus is a major contributor to hip extension, abduction, and external rotation strength. Several researchers have reported that a decreased hip muscle strength (abduction and external rotation) is associated with risk factors for ACL injury (e.g., greater knee valgus angle, knee valgus moment, and loss of frontal postural stability) among others [27,28,29]. Prospective studies have shown that small hip abductors and external rotator muscle strength predict the risk of ACL injury [30]. Our study found that female badminton players exhibited greater gluteus maximus muscle activity and greater valgus angle compared to males. In studies addressing the triad of hip muscle strength, gluteus maximus muscle activity, and valgus angle, it was shown that small hip muscle strength and large gluteus maximus muscle activity was associated with a large valgus angle [16,27,31,32]. They concluded that when there is insufficient gluteus muscle strength to maintain dynamic valgus, participants can compensate by sending more neural signals to try to activate as much gluteus muscle tissue as possible [27]. This suggests that individuals with low gluteus strength are mechanically disadvantaged in resisting frontal plane knee motion and may be more prone to hip muscle tissue fatigue [33]. Anne Benjaminse et al. demonstrated in a review study the important effect of fatigue on ACL injury risk during exercise [34]. This seems to imply that the large gluteus maximus activation strategy and knee valgus angle exhibited by female badminton players at high risk of injury are associated with possibly weaker muscle strength in their hips. Therefore, if the generation of force required to maintain stability is achieved by strengthening the hip muscles and less neural drive is required, the risk of fatigue and ACL injury could be reduced. In addition, strengthening the hip muscles may improve neural efficiency and reduce the need for gluteus maximus muscle activity, thereby delaying the onset of fatigue. Notably, we did not test hip strength in badminton players; therefore, further studies could identify this risk factor through prospective studies with badminton players and demonstrate its effectiveness by designing intervention experiments to improve the precise validity of our injury prevention strategy for people at high risk of an ACL injury.

It is well known that the distal quadriceps are connected to the anterior proximal tibia via the patellar ligament, and when smaller knee angles, specifically the patellar tendon-tibial axis angle [35] and the ACL elevation angle [36] (the angle between the ACL and the tibial plateau) simultaneously increases, this implies an increase in the horizontal component of the knee extension forces generated by the quadriceps contraction, i.e., an increase in the anterior proximal tibial shear force. Our study found that the activity level of the rectus femoris muscle was higher in female badminton players, which is consistent with the results of previous studies on the landing preparation phase by Chappell, J.D. et al. [37] and Zazulak, B.T. et al. [38]. In previous studies, the rectus femoris dominant strategy was considered to be the direct cause of cruciate ligament injuries. It is worth noting that the function of the rectus femoris is thought to be related to the control of hip flexion and extension, and knee flexion and extension; however, although female badminton players in our study showed higher levels of rectus femoris activity, no differences were found between the hip and knee in sagittal plane angles, which may mean that this phenomenon cannot be explained in terms of lower limb segments alone. Earlier studies have shown that muscle activity in the lower limbs can be altered by changes in trunk and pelvis position [39,40]. This dominant rectus femoris strategy can be explained by whole body movement patterns and is an adaptive response to the posture observed in women during landing. Therefore, future studies investigating gender differences in ACL injuries during functional tasks should link local knee stability to whole body biomechanical factors. Further research should investigate the influence of trunk kinematics and muscle activity on risk factors for ACL injury in women.

During the landing preparation phase, female badminton players showed greater activity of both medial and lateral gastrocnemius muscles when performing the single-leg landing task, compared to male badminton players. This gender difference in neuromuscular control strategy has been reported in previous landing tasks [12,41]. Based on models and in vivo studies, some arguments have linked gastrocnemius contraction to injury risk factors in ACL [9,42]. Navacchia, A. et al. [9] estimated knee anterior shear force during the landing task by EMG-based musculoskeletal models, and their results showed that the gastrocnemius was the largest muscle contributor to peak tibial anterior shear force. Adouni, M. et al. [42] examined the activity of individual gastrocnemius muscles at different knee angles (0–90 degrees), all of which greatly increased ACL stress. A study that investigated the effect of gastrocnemius stimulation levels on tibial anterior displacement by ultrasonography demonstrates that gastrocnemius increases tibial anterior displacement when activated regardless of the stimulation level; thus, supporting gastrocnemius’ role as an antagonist of the ACL [43]. They suggest that this association of the gastrocnemius with the ACL may be because the gastrocnemius is located posteriorly to the knee joint, proximally at the femur originating at the medial and lateral femoral epicondyles and distally ending at the heel tuberosity, spanning the posteriorly protruding tibial plateau. Furthermore, when the larger gastrocnemius is activated, the increased muscle volume generating compressive forces will result in increased anterior shear forces and increased anterior displacement of the tibia [43]. Another in vivo study showed that when the gastrocnemius muscle contracts, this coupling will make the strain on the ACL greater when combined with the knee valgus angle, and in our study, female badminton players exhibited this combination. In opposition to this, some arguments suggest that gastrocnemius activity plays a protective role in the ACL [44,45]. Morgan, K.D. et al. [45] and Ali, N. et al. [44] studied the single-leg landing task in the same way as multi-musculoskeletal modeling, and their results showed that an increase in gastrocnemius muscle strength was associated with a decrease in stress in tibial anterior shear or ACL. Additionally, this difference may be related to the way of modeling they used.

Furthermore, our study discovered that there was variability in the co-contraction of the medial and lateral gastrocnemius muscles with female badminton players showing a smaller ratio of co-activation of the medial and lateral gastrocnemius muscles, compared to males. This is similar to the results of Beaulieu, M.L. et al. [12]. In terms of the frontal plane of the knee, the medial and lateral muscles play opposite roles to each other with the medial gastrocnemius more inclined to produce varus angulation and varus loading of the knee, whereas the lateral gastrocnemius is more inclined to produce valgus angulation and valgus loading [46,47]. The co-contraction of both helps to balance the varus and valgus moments and maintain frontal plane stability, and a greater co-contraction ratio of the medial and lateral gastrocnemius in males provides assistance in preventing greater valgus loads and angles, which helps to maintain knee stability and may be more beneficial in preventing the development of risk factors for ACL injury. Conversely, this co-contraction pattern makes female badminton players potentially at greater risk of developing ACL injuries, compared to males.

There are several limitations to this study. Firstly, there are limitations to the data collection process for both EMG and 3D motion analysis [48,49]. Model measurement errors, including misaligned knee markers, errors in skin movement artefacts, and 3D analysis of EMG signals are highly complex, stochastic, and susceptible to inherent individual factors that may affect the results of the data. However, although 3D motion analysis systems have their limitations, their widespread use is why we believe they are still a valid means of analysis. To reduce the effects of the high variability inherent in EMG signals, we analyzed more trial data for each participant; thus, ensuring a higher level of confidence in our findings. Secondly, the high-risk task of a cruciate ligament injury in badminton players has been performed in a laboratory setting and although not a complete substitute for a field setting, we modeled this potentially injurious action as realistically as possible, which allowed us to isolate more credible information, compared to traditional studies.

## 5. Conclusions

During the landing preparation phase of the badminton single-leg landing task, there were significant gender differences in neuromuscular control (muscle activity patterns, movement patterns) between badminton players. Female badminton players exhibit neuromuscular control strategies that may be inadequate for ACL protection that may be a potential risk factor for a high incidence of ACL injury. In the future, when developing programs to prevent ACL injuries in female badminton players could consider targeting the optimization of neuromuscular control during the pre-landing preparation phase.

## Figures and Tables

**Table 1:** Muscle names, electrode site, and specific positions and maneuver for each muscle during the maximum voluntary isometric contraction (MVC) test.

Muscle	Electrode Site	Position	MVC Test Maneuver
Gluteus maxim (GMAX)	Lateral 80% of the line between the midpoint of the sacrum and the greater trochanter of the femur.	Prone	The stretch strap is fixed to the posterior side of the distal thigh and hip extension is performed with the knee in 90 degrees of flexion.
Gluteus medius (GMED)	Upper 20% of the line between the greater trochanter of the femur and the highest point of the iliac spine.	Lateral prone	The stretch strap is fixed to the lateral side of the distal thigh and hip abduction is performed with the knee flexed at 90 degrees.
Rectus femoris (RF)	Upper 40% of the line between the superior patella and the anterior superior iliac spine.	Sitting	Knee flexion at 90 degrees, the stretch strap is fixed on the anterior side of the distal lower leg, perform knee extension.
Medial hamstrings (MH)	Lower 80% of the line of the ischial tuberosity with the medial popliteal crease.	Prone	Knee flexion 45 degrees, the stretch strap is fixed on the back of the distal lower leg, internal rotation of the lower leg and perform knee flexion.
Lateral hamstrings (LH)	Lower 80% of the line of the ischial tuberosity to the lateral popliteal crease.	Prone	Knee flexion 45 degrees, the stretch strap is fixed on the back of the distal lower leg, external rotation of the lower leg and perform knee flexion.
Medial gastrocnemius (MG)	Upper 85% of the medial Achilles tendon in line with the medial popliteal crease	Prone	Knee extension, stretch strap fixed on the forefoot, internal rotation of the lower leg and perform plantarflexion.
Lateral gastrocnemius (LG)	Upper 85% of the line connecting the lateral Achilles tendon to the lateral popliteal crease	Prone	Knee extension, stretch strap fixed on the forefoot, external rotation of the lower leg and perform plantarflexion.

**Table 2:** Means and standard deviations (degrees) of hip-knee-ankle joint angles during the landing preparation phase of the single-leg landing task in badminton. * Represents statistically significant differences.

Variables	Male	Female	F	*p*-Value
Hip				
Flexion (+)/extension (−)	11.26 ± 8.31	11.83 ± 8.60	0.018	0.8951
Abduction (+)/adduction (−)	36.33 ± 4.37	39.86 ± 5.59	1.981	0.1811
External (+)/internal rotation (−)	15.84 ± 5.32	17.94 ± 13.87	0.160	0.6950
Knee				
Flexion (+)//extension (−)	19.47 ± 6.42	18.08 ± 7.33	0.163	0.6924
Valgus (+)//varus (−)	1.72 ± 3.20	6.27 ± 2.75	9.284	0.0087 *
External (+)/internal rotation (−)	−4.39 ± 4.67	0.15 ± 4.29	4.105	0.0622
Ankle				
Dorsiflexion (+)/plantar flexion (−)	−39.92 ± 9.25	−33.39 ± 6.45	2.675	0.1242
Eversion (+)/inversion (−)	0.47 ± 3.45	−0.28 ± 4.32	0.058	0.8141
Abduction (+)/adduction (−)	16.98 ± 4.63	23.16 ± 6.75	4.395	0.0562

## Data Availability

The data presented in this study are available on request from the corresponding author.
